# The Effects of Silica on the Properties of Vitreous Enamels

**DOI:** 10.3390/ma12020248

**Published:** 2019-01-13

**Authors:** Signo T. Reis, Mike Koenigstein, Liang Fan, Genda Chen, Luka Pavić, Andrea Moguš-Milanković

**Affiliations:** 1 Roesch Inc., 100 North 24th Street, Belleville, IL 62226, USA; MKoenigstein@roeschinc.com; 2 Department of Materials Science & Engineering, Missouri University of Science and Technology, Rolla, MO 65409-0340, USA; 3 Department of Civil, Architectural, and Environmental Engineering, Missouri University of Science and Technology, Rolla, MO 65409-0030, USA; lf7h2@mst.edu (L.F.); gchen@mst.edu (G.C.); 4 Ruđer Bošković Institute, Division of Materials Chemistry, Bijenička c. 54, 10000 Zagreb, Croatia; lpavic@irb.hr (L.P.); mogus@irb.hr (A.M.-M.)

**Keywords:** glass enamel, acid resistant, silica, Raman spectroscopy

## Abstract

Ground coat enamels for low carbon steel that contain silica as a mill addition have been developed to study the changes of their properties. Acid-resistant commercial enamel where silica addition was varied from 0 to 10.0 wt % was used for this investigation. The effects of the addition on the corrosion resistance, thermal properties, electrical properties, and mechanical adherence of the enamel to low carbon steel were studied. The corrosion resistance of the steel enameled coupons was tested using a salt spray (fog) apparatus for time periods reaching 168 h at room temperature. It was found that, although the density was not affected, the adherence decreased with an increase in silica content. As expected, the silica addition decreased the coefficient of thermal expansion, which is directly related to the increasing stress between the glass and steel in accordance with the adherence results. A mill addition of 7.5 wt% of silica to the samples was sufficient to obtain adequate enamel adherence and good corrosion resistance. Furthermore, the addition of silica influenced the electrical conductivity and dielectric permittivity measurements at room temperature and the conductivity measured in a wide frequency range (1 Hz–1 MHz). The dielectric permittivity measured at 1 MHz showed decrease after the addition of up to 7.5 wt% of silica.

## 1. Introduction

Enamels are deposited onto metallic surfaces to improve their corrosion resistance [1]. The enamel produces a vitreous coating on the metal articles that protects the metal from the effect of liquids, gaseous, and solid reactants [2]. The extensive range of vitreous enamel compositions available and the possibility of tailoring their protective properties make them suitable for use in a wide variety of service conditions and on different substrates [3]. The new formulation of enamel coatings should improve properties such as strength, gloss, surface smoothness, acid protection, etc. [2]. Corrosion or chemical resistance in service is a major consideration for the final customers and should lead to a proper formulation of the enamel frit. In acid media, where the reaction rate of the ion exchange process predominates, the dissolution processes strongly depends on the composition of the coating [4]. 

Over 90% of the aqueous corrosion of coatings can be accounted for by considering three factors: the time of exposure, pH of the medium, and the temperature. The rate of exchange of hydronium ions with alkali ions from the glass surface in acid environments has been studied by many authors [3,4,5,6]. Many acid-resistant enamel formulations are described in the literature [7,8]. The additions in glass frits usually improve the corrosion resistance and change the glass enamel performance by decreasing the adherence of the enamel to the substrate [9,10,11]. This factor is to be taken into consideration when designing a corrosion-resistant enamel. Recently, various formulations of corrosion-protected enamels have been investigated to protect reinforcing steel from corrosion in concrete as well as to improve the cement-to-steel bonding [11].

The present work aims to investigate and discuss the role of silica addition in corrosion resistance in saltwater, the mechanical adherence of enamel to low-carbon steel, and the thermal and electrical properties of a typical commercial boron-silicate glass enamel. 

## 2. Experimental

### 2.1. Sample Preparation

The steel substrate used in this study consisted of low carbon steel supplied by Kloecker Metals Corp. (Roswell, GA, USA, http://www.kloecknermetals.com/Home.aspx). Table 1 shows the chemical components of this steel in wt%. The steel sheet was cut into discs of 2 mm thickness by 78 mm in diameter. The basic enamel recipe used was a typical enamel frit with good corrosion-resistance properties. The enamel frit was basically a boron-alumina silicate containing alkalines to match the steel thermal expansion and adherent oxides such as cobalt, nickel, manganese, and copper to improve the bonding to the steel. The frit composition analyzed by inductively coupled plasma atomic emission spectroscopy (ICP-AES, Bureau Veritas, Paris, France, http://acmelab.com/services) is shown in Table 2.

Trials were conducted by increasing the amount of quartz in the mill addition from 0 wt% to 10.0 wt%, under the hypothesis that the increase in silica content would have a positive effect on the acid resistance of the enamel. The dry powder of the enamel frit with the mill additions was weighed accurately, mixed with silica and ground in a ball mill to obtain a powder of size < 200 mesh. Table 3 shows the sample formulation.

**Figure 1:**
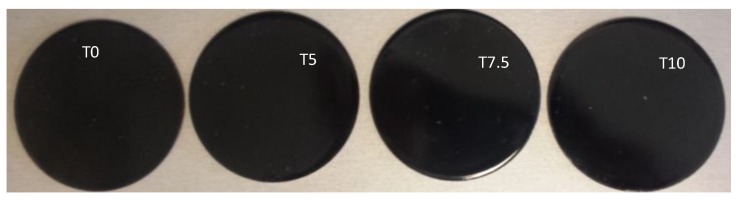
Digital pictures of the ground coat enamel with silica addition fired at 840 °C for 4 min in air.

The slurry was prepared by mixing the milled frit with 40 wt% water. This mixture was then agitated for 10 min, and then applied by wet spraying onto the metal surface. Prior to application, the steel coupons were cleaned using alkaline soap in warm water at 80 °C for 5 min. This was followed by hot-water and then cold-water rinsing. After the wet spraying application, the samples were dried at 100 °C for 10 min and fired to 840 °C for 4 min in a muffle furnace. Measurement of the enamel layer thickness using the Mini Test 650 coating thickness gauge (ElektroPhysik, Cedarhurst, NY, USA, https://www.elektrophysik.com) showed that it ranged from 150 to 200 μm. Figure 1 shows digital pictures of the samples after firing with no difference in appearance among the samples.

### 2.2. XRD, Density and Dilatometric Analysis

Room temperature X-ray diffractograms (XRD) for enamels coated on steel coupons were collected using an X-ray diffractometer, Scintag XDS2000X (Franklin, MA, USA). 

Dilatometric analysis (Orton dilatometer model 1600D, Westerville, OH, USA) measurements were performed in flowing synthetic air at a heating rate of 10 °C/min to determine the softening temperature (T_S_) and coefficient of thermal expansion of the glass enamel. The samples of glass enamel were produced by melting batches from the mixing in accordance with Table 2 in alumina crucibles in air at 1250 °C for 1 h. The melted sample was quenched in air by pouring it into a 1 × 1 × 5 cm^3^ steel mold. The samples were transferred into a furnace and annealed at 450 °C for 4 h. The glass enamel density was measured using the Archimedes method with distilled water as the buoyancy liquid.

### 2.3. Procedures for the Impact Resistance and Corrosion Test

The impact resistance test was performed in accordance with the ISO4532 standard (load of 20 N) to check the degree of bonding between the fused vitreous enamel and the metallic substrate. The enamel performance was examined by using Hirox microscope (Hirox-USA, Hackensack, NJ, USA).

The corrosion test by salt spray exposition was performed according to the ASTM B117-16 to obtain corrosion resistance information relative to specimens of metals coated with porcelain enamels. The test consists of a one week (168 h) long exposure of the specimen surface to a fog of salt solution prepared by dissolving 1 part by mass of sodium chloride in 95 parts of deionized water with chamber temperature maintained at 35 °C. Prior to the test, the edge of each sample was covered with marine epoxy to avoid salt solution penetration into the steel. After one week of exposure, the samples were removed from the chamber, cleaned with deionized water, and dried in an oven at 80 °C overnight. The dried samples were weighed to determine the weight loss due to corrosion. A precision balance with precision of 0.0001 g was used for weight measurements. Each sample was tested twice to decrease experiment error. 

### 2.4. Electrical Measurements

**Figure 2:**
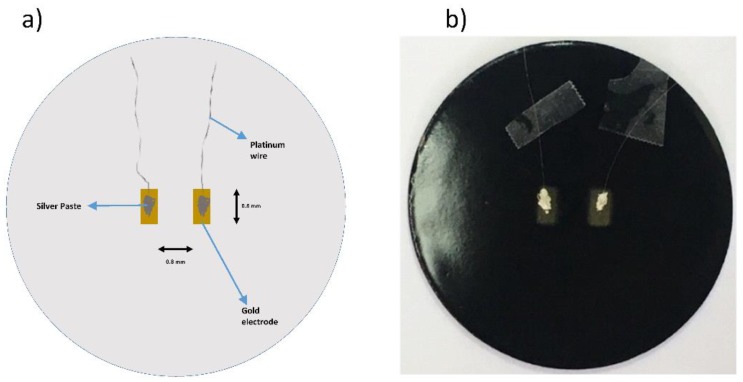
(**a**) The setup diagram for the electrical measurements, (**b**) and a digital picture of the sample with the prepared setup.

For the electrical contacts, gold electrodes (4 mm × 8 mm) separated from each other by 8 mm were deposited on the sample surface (see Figure 2) using a Sputter Coater SC7620 (Quorum Technologies Ltd., Laughton, Lewes, UK). Platinum wires were attached on the surface of gold pads with silver paste to make a connection with instrument cell. The electrical and dielectric surface properties of samples were analyzed by measuring complex impedance using an impedance analyzer (Novocontrol Alpha-AN Dielectric Spectrometer [12], Novocontrol Technologies GmbH & Co. KG, Aubachstr, Montabaur, Germany) in the frequency range from 1 Hz to 1 MHz at room temperature.

## 3. Results

### 3.1. XRD

**Figure 3:**
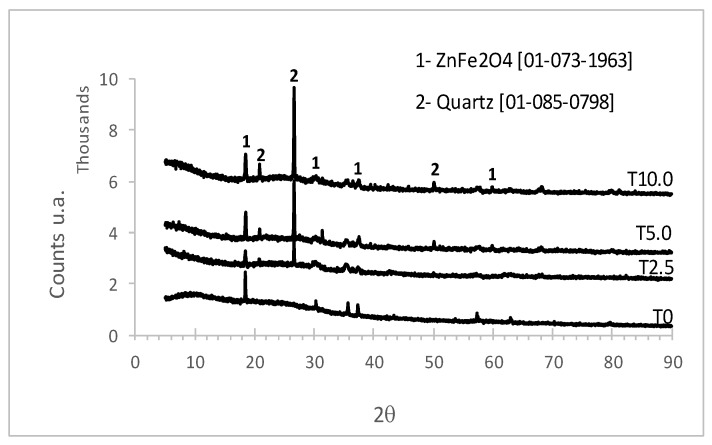
XRD of the surface of low-carbon steel coated with glass enamel samples fired at 850 °C for 4 min.

The XRD measurement results obtained on the glass enamel surface for all samples are shown in Figure 3. Along with a wide halo, characteristic for the amorphous state, diffraction lines of the ZnFe_2_O_4_ (PDF 01-073-1963) crystalline phase—probably due to a reaction with the milling balls—were detected. For samples containing a higher silica content, T2.5 to T10.0, the intensity of the diffraction lines that correspond to quartz (Powder Diffraction File (PDF) 01-085-0798) were more pronounced, indicating a higher amount of quartz crystalline phase present in those samples.

### 3.2. Density and Dilatometric Analysis

**Figure 4:**
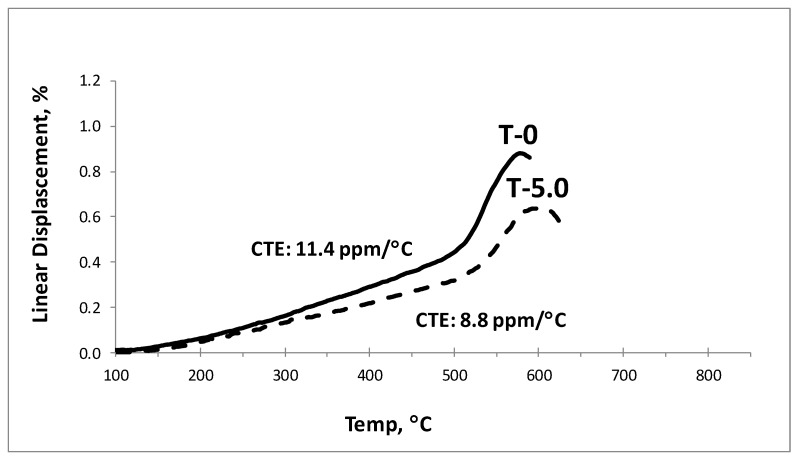
Dilatometric analysis of the T0 and T5.0 glass enamels.

As shown in Table 4, there was no significant increase in density with the addition of silica from 0 wt% (T0) to 10.0 wt% (T10). However, the glass transition (T_g_), softening temperature (T_s_), and coefficient of thermal expansion (CTE) calculated from dilatometric analysis (see Table 4 and Figure 4) showed considerable changes with silica addition. The glass transition temperature, T_g_, increased from 511 °C to 535 °C. The softening temperature, Ts, also increased from 578 °C to 606 °C for the T0 to T10 samples, whereas the CTE decreased from 11.4 ppm/°C to 7.4 ppm/°C.

### 3.3. Effect of Silica on the Impact Resistance

**Figure 5:**
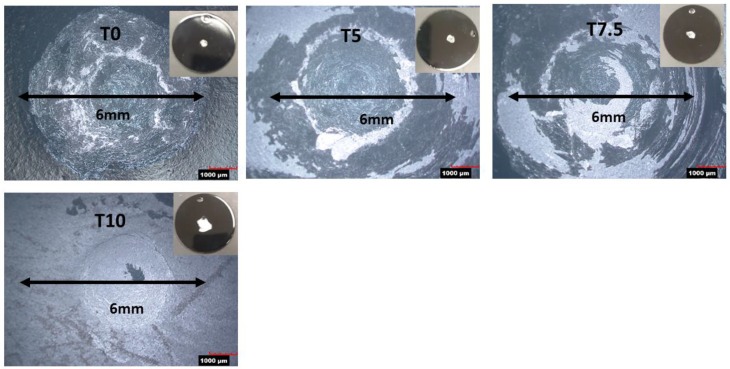
Micro-photographs of the impact centers of the enamel coatings of samples T0, T5.0, T7.5 and T10.0 after the impact test.

Figure 5 shows images of the impact centers of the enameled low carbon steel samples after the impact resistance tests. The impact surface of sample T0 (0 wt% silica) and T5 (5.0 wt% silica) were largely covered with enamel, which is characterized as having excellent adherence in accordance with the ISO4532A. The impact surface of sample T7.5 (7.5 wt% silica) was still largely covered with enamel but with considerable bare areas, classified as moderate adherence. However, specimen T10 (10.0 wt% silica) was bare to a large extent, indicating poor adherence. The results indicate that the adherence of the enamel coating to the low carbon steel substrate decreased with the silica addition.

### 3.4. Effect of Silica on the Corrosion Resistance

**Figure 6:**
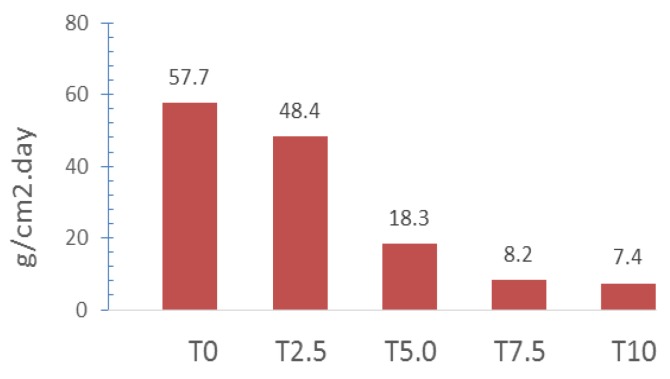
Weight loss of samples exposed to salt spray over the course of seven days.

The degree of attack by a salt spray (fog) provides a good indication of the susceptibility to corrosion of a porcelain enamel coating in natural environments. The mass loss of steel in the salt spray practice is dependent upon the area of steel exposed, the temperature, time of exposure, salt solution makeup and purity, pH, spray conditions, and the metallurgy of the steel. Figure 6 shows the effect of the percent of silica on the variation of the percent loss in weight with silica addition calculated as loss in weight per unit area per day. It can be seen from Figure 6 that the specific weight loss (g/cm^2^ day) decreased in all cases, as the percent of silica addition increased. There was no visual discoloration of the samples after the test.

### 3.5. Effect of Silica on the Electrical Properties

**Figure 7:**
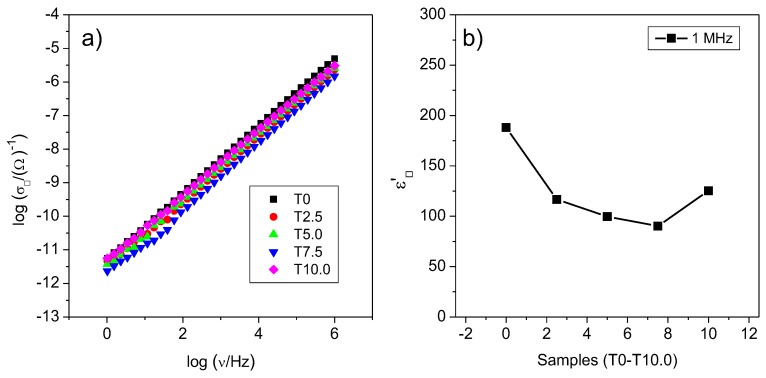
(**a**) Conductivity spectra for all samples, (**b**) and dielectric permittivity as a function of silica addition measured at room temperature.

Figure 7 shows the effect of the addition of silica on the conductivity and dielectric permittivity measured at room temperature. With the addition of silica, the conductivity measured in a wide frequency range (1 Hz–1 MHz) and the dielectric permittivity measured at 1 MHz decreased up to 7.5 wt%. The further addition of silica resulted in a slight increase of both conductivity and dielectric permittivity.

## 4. Discussion

Corrosion resistant enamel with the composition shown in Table 2 and a series of samples with varied amounts of silica from 0 to 10.0 wt% (see Table 3) were prepared. When adding silica up to 10.0 wt%, a considerable effect on the glass transition, softening temperature and CTE was observed. It was found that silica addition decreased the CTE, which is directly related to the increasing stress between the glass and steel. This consequently affects the impact resistance of the enameled metal substrate and the enamel adherence with the steel. In accordance with ISO4532A, the silica addition decreases the enamel adherence to steel because of the increase of thermal stress between the enamel and steel during the cooling. The salt spray test was used to test the resistance of enamel frits to chemical attack. It was found that the extent of the attack decreased with an increase in silica content. The protective effect of the enamel, regardless of the extent or the rate of attack, began to show appreciably after a 5.0 wt% silica addition. This effect stabilized after the addition of 7.5–10.0 wt% silica. It was also found that the weight loss rapidly decreased up to 7.5 wt% silica content, whereas a much slower decrease was observed in samples containing 7.5 wt% and 10.0 wt% of silica. Therefore, it can be concluded that it takes at least 7.5 wt% of silica addition in the mill to achieve a reasonable corrosion resistance. On the other hand, silica levels higher than 7.5 wt% did not produce any appreciable additional corrosion resistance.

The addition of silica influenced the electrical conductivity and dielectric permittivity measurements at room temperature. With the increasing silica content, the conductivity at all measured frequencies (1 Hz–1 MHz) and dielectric permittivity measured at 1 MHz showed a decrease of up to 7.5 wt%. Such behavior can be explained as follows. It is known that the initial addition of oxide into dielectric materials can decrease the electrical conductivity and dielectric parameters of a boron-alumina-silicate enamel. Usually such a decrease in conductivity is related to the initial structural disorder while further oxide addition stabilizes the structure network, resulting in an easier transport of charge carriers through conduction pathways. It is worth mentioning that the observed slight changes in electrical conductivity relate to a slight difference in frit enamel composition as various amounts of silica, 0–10 wt% were added. The obtained electrical data could be correlated with the resistance of enamel frits to the chemical attack showing a protective effect that stabilized weight loss at about 7.5 w% of silica. It seems that in the compositional range up to 7.5 wt% of silica a disordered structural network was present. 

Furthermore, the addition of silica affected the formation of quartz crystallites on the surface of enamel layer, which is consistent with the XRD analysis, see Figure 3. As expected, the formation of randomly-distributed quartz crystallites decreased electrical conductivity and dielectric permittivity. In crystallized iron phosphate glasses, the formation Fe_3_(P_2_O_7_)_2_ crystallites causes a drop of total electrical conductivity due to the blocking of the conduction pathways for the electron hopping between Fe sites at the crystal/glass matrix interfaces [13]. On the other hand, with the further addition of silica up to 10.0 wt% the crystallites became more uniformly distributed, leading to the formation of easier conductivity pathways. However, it should be mentioned that changes in the electrical and dielectric properties related to the formation of crystallite networks on the enamel surface corresponded well with the corrosion test, i.e., with the resistance to the chemical attack performed using ASTM B117-16.

## 5. Conclusions

Corrosion resistant enamel was prepared with various percentages of silica addition. The addition of silica up to 10.0 wt% content showed a considerable effect on the glass transition, softening temperature, and coefficient of thermal expansion. The silica addition decreased the coefficient of thermal expansion, which is directly related to the thermal stress created between the glass and steel and consequently affects both the impact resistance of the enameled metal substrate and the enamel adherence to the steel. It was also found that the extent of corrosion attack decreased with the increasing silica content. The protective effect stabilized at the addition of 7.5–10.0 wt% of silica. This means that it took at least 7.5 wt% of silica addition in mill to achieve a reasonable corrosion resistance. The samples containing up to 7.5 wt% of silica exhibited a decrease, whereas the addition of 10.0 wt% of silica showed a slow increase in the electrical conductivity and dielectric permittivity due to the uniformly formed crystallite network on the enamel surface. These results were in an excellent accordance with the corrosion test, which led to the conclusion that the addition of 7.5 wt% of silica is sufficient to obtain adequate enamel adherence and good corrosion resistance.

## Figures and Tables

**Table 1:** Chemical analysis of the steel substrate.

Elements	C	Mn	P	S	Si	Cu	Ni	Cr	Al	N
wt%	0.04	0.18	0.01	0.005	0.013	0.015	0.01	0.03	0.04	0.04

Fe as balance.

**Table 2:** Enamel frit composition obtained by ICP-AES analysis.

Oxides	SiO_2_	B_2_O_3_	Na_2_O	Al_2_O_3_	CaO	TiO_2_	MnO	Fe_2_O_3_	CoO	NiO	CuO	ZnO	ZrO_2_
wt%	53.9	12.5	12.7	1.0	3.8	1.6	3.9	1.3	1.3	2.6	1.4	2.5	0.5

**Table 3:** Sample formulation.

Sample ID	Glass Frit (wt%)	Silica Addition (wt%)
T0	100	0
T2.5	97.5	2.5
T5.0	95.0	5.0
T7.5	92.5	7.5
T10.0	90.0	10.0

**Table 4:** Density and thermal properties of enamel glasses with silica addition.

Sample ID	Density (g/cm^3^)	T_g_ (°C)	T_s_ (°C)	CTE (200 to 400) (ppm/°C)
T0	2.56 ± 0.02	511	578	11.4
T2.5	2.63 ± 0.01	514	592	8.8
T5.0	2.64 ± 0.02	530	603	8.3
T7.5	2.62 ± 0.02	531	604	7.3
T10.0	2.63 ± 0.02	535	606	7.4
